# Supplementary material for: Transcription start site profiling uncovers divergent transcription and enhancer-associated RNAs in Drosophila melanogaster
Source: BMC Genomics. 2018 Feb 21;19:157. doi: 10.1186/s12864-018-4510-7 (PMC5822475; doi:10.1186/s12864-018-4510-7)
Supplement: Supplementary file 1 — Containing Figures S1 thru S7. (PDF 1904 kb) [file 12864_2018_4510_MOESM1_ESM.pdf]

Additional File 1: Containing Figures S1 thru S7

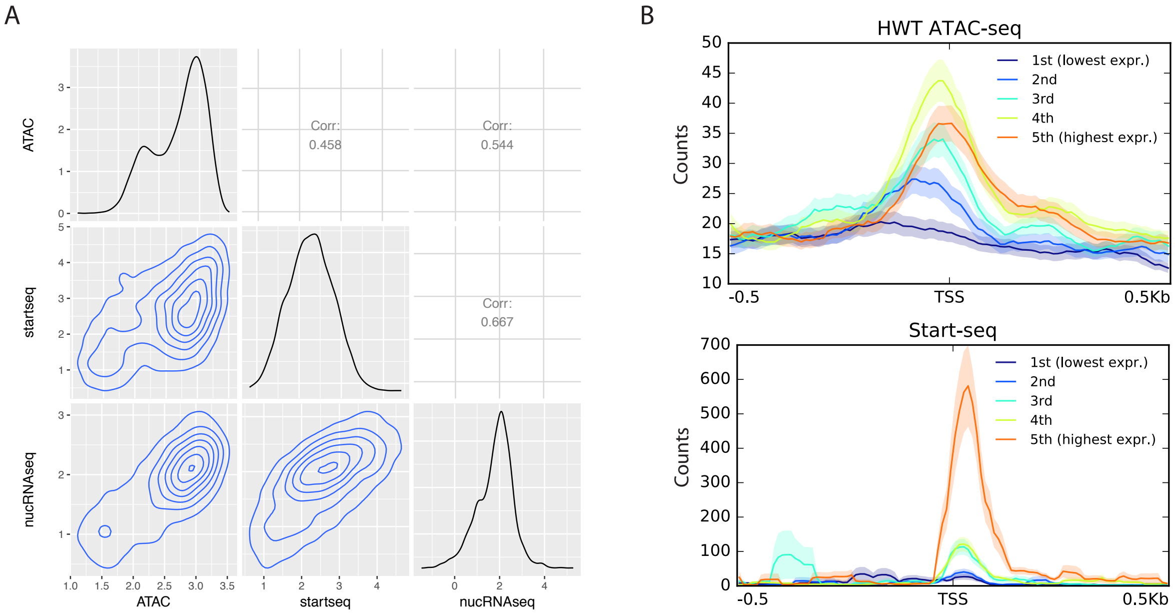

Figure S1: A) Pairwise comparisons of ATAC-seq, Start-seq, and nuclear RNA-seq for annotated coding genes. Boxes on diagonal show density plots of log signal enrichment for each assay. Boxes in on upper right show correlation values for each pairwise comparison. Boxes on lower left show contour plots representing scatterplots of log enrichment for each pairwise comparison. B) ATAC-seq and Start-seq signal accumulation at genes stratified into quintiles by nuclear RNA-seq signal. As is evident from lower ATAC-seq signal in the highest expression quintile, and by low stratification of lower quintiles by Start-seq, ATAC-seq and Start-seq are inconsistently correlated with nuclear RNA-seq.

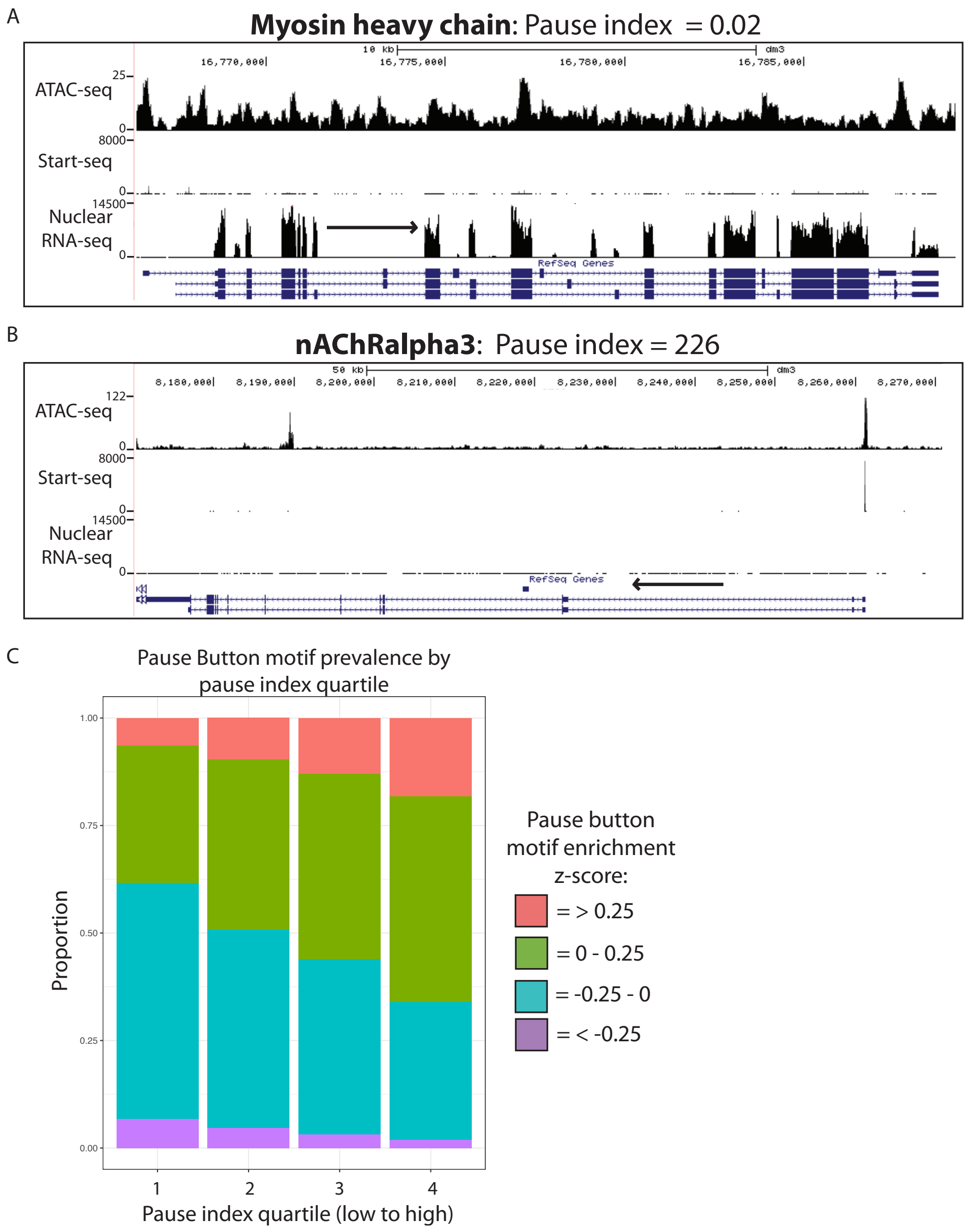

Figure S2: A) Representative browser window example of gene with low pause index. Note low Start-seq and high nuclear RNA-seq values. B) Representative browser window example of gene with high pause index. Note high Start-seq and low nuclear RNA-seq values. C) Enrichment of "Pause Button" (PB) motif based on pause index (PI) quartiles. Bars are partitioned based on z-score of highest log-odds score for enrichment of the PB motif in every nuTSS in the cohort. High PI quartile (right) disproportionately contains nuTSSs with high PB z-scores.

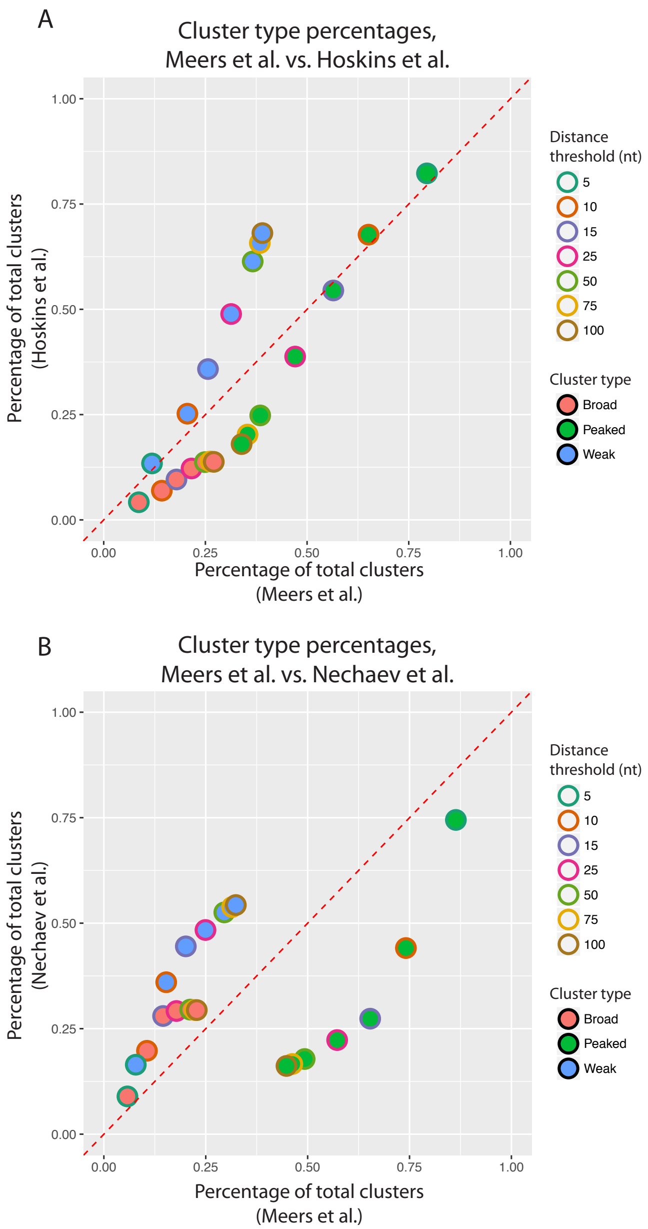

Figure S3: Comparison of the percentage of peaked (green points), broad (red), and weak (blue) clusters detected in this study (x-axis) as compared to Hoskins et al. [13] (A) or Nechaev et al. [19] (B) (y-axis). For each comparison, datasets were subsampled at equal read depth, signal was assigned to peaks using the same method, and the cluster distance threshold was varied (as denoted by colored point borders).

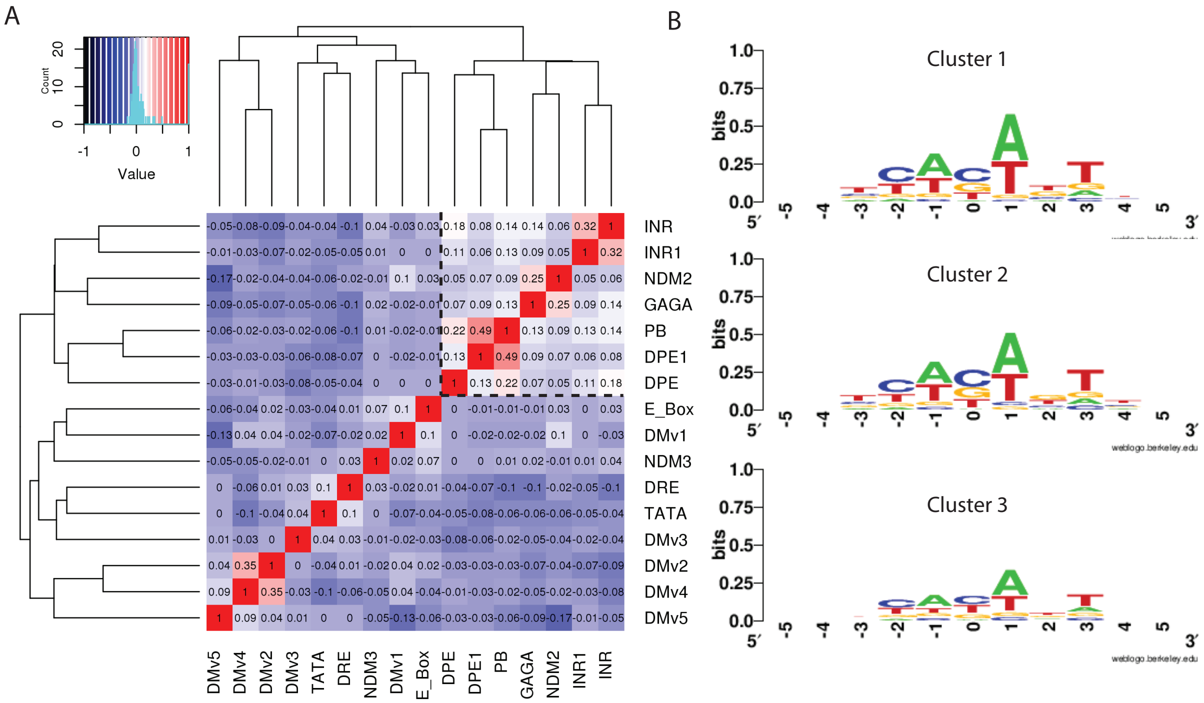

Figure S4: A) Correlation matrix of enrichment of 16 motifs used in clustering of obsTSSs. Dashed black lines denote group motifs co-occurring more frequently than others. B) Consensus motifs at obsTSSs based on motif-derived clustering. Cluster 1 resembles INR motif (TCAGT) from position -1 to 3, whereas other groups have lower sequence information content.

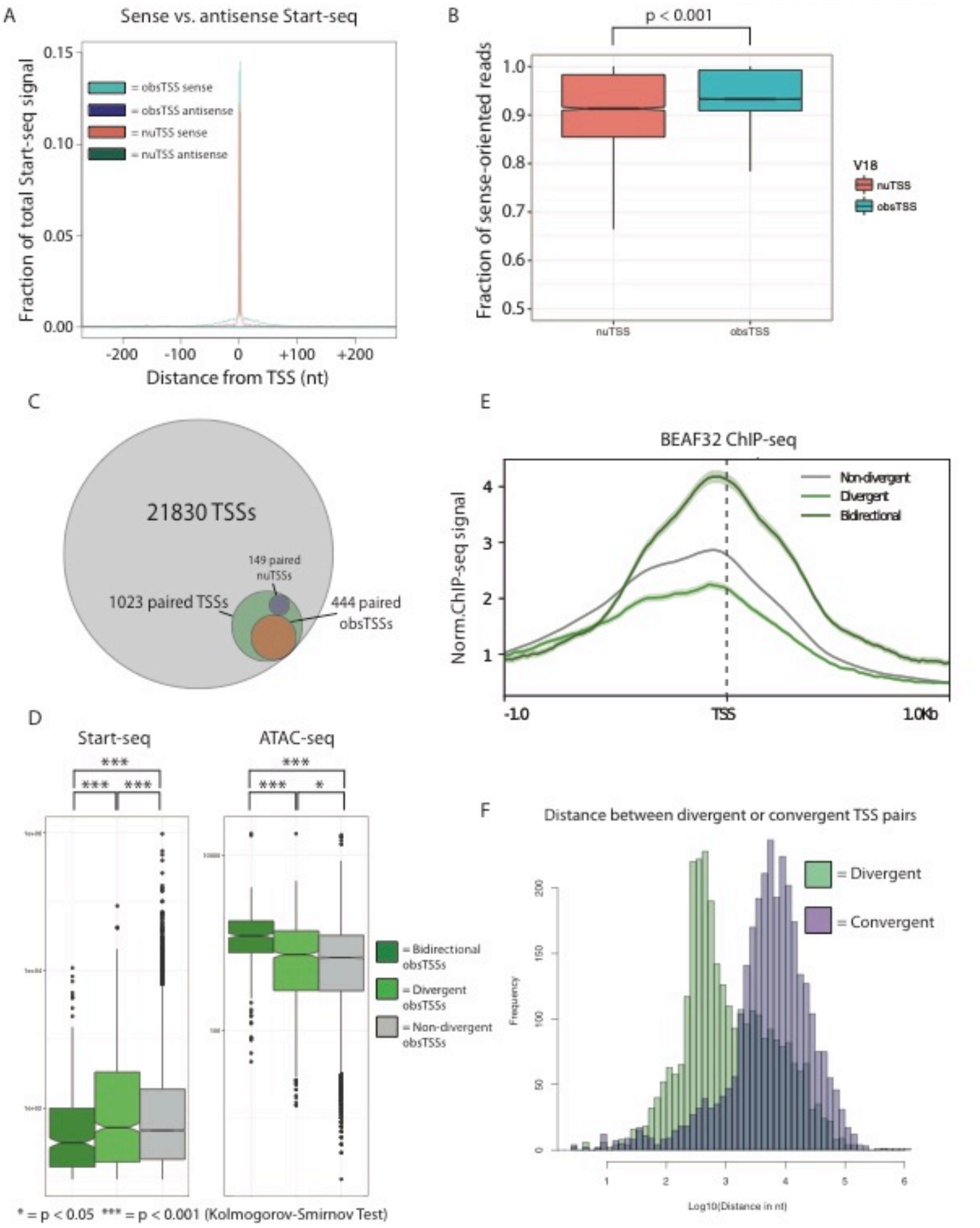

Figure S5: A) Metaplot of sense or antisense Start-seq signal mapping in a 500nt window around obsTSSs (teal and purple, respectively) or nuTSSs (salmon and green, respectively). B) Boxplot describing fraction of sense-oriented reads mapping in a 400nt window around nuTSSs (salmon) or obsTSSs (teal). C) Venn diagram describing representation of paired TSSs within high-confidence TSSs detected by Start-seq. D) Boxplots describing Start-seq (left) and ATAC-seq (right) signal corresponding to non-divergent obsTSSs (grey), divergent obsTSSs (i.e. obsTSSs paired with nuTSS, light green), and bidirectional obsTSSs (dark green). E) Metaplot describing BEAF32 ChIP-seq signal mapping in a 2kb window surrounding non-divergent (grey), divergent (light green), and bidirectional (dark green) obsTSSs. F) Histograms describing the distributions of distance between divergent (green) and convergent (purple) pairs of nearest neighbor obsTSSs oriented in opposite directions.

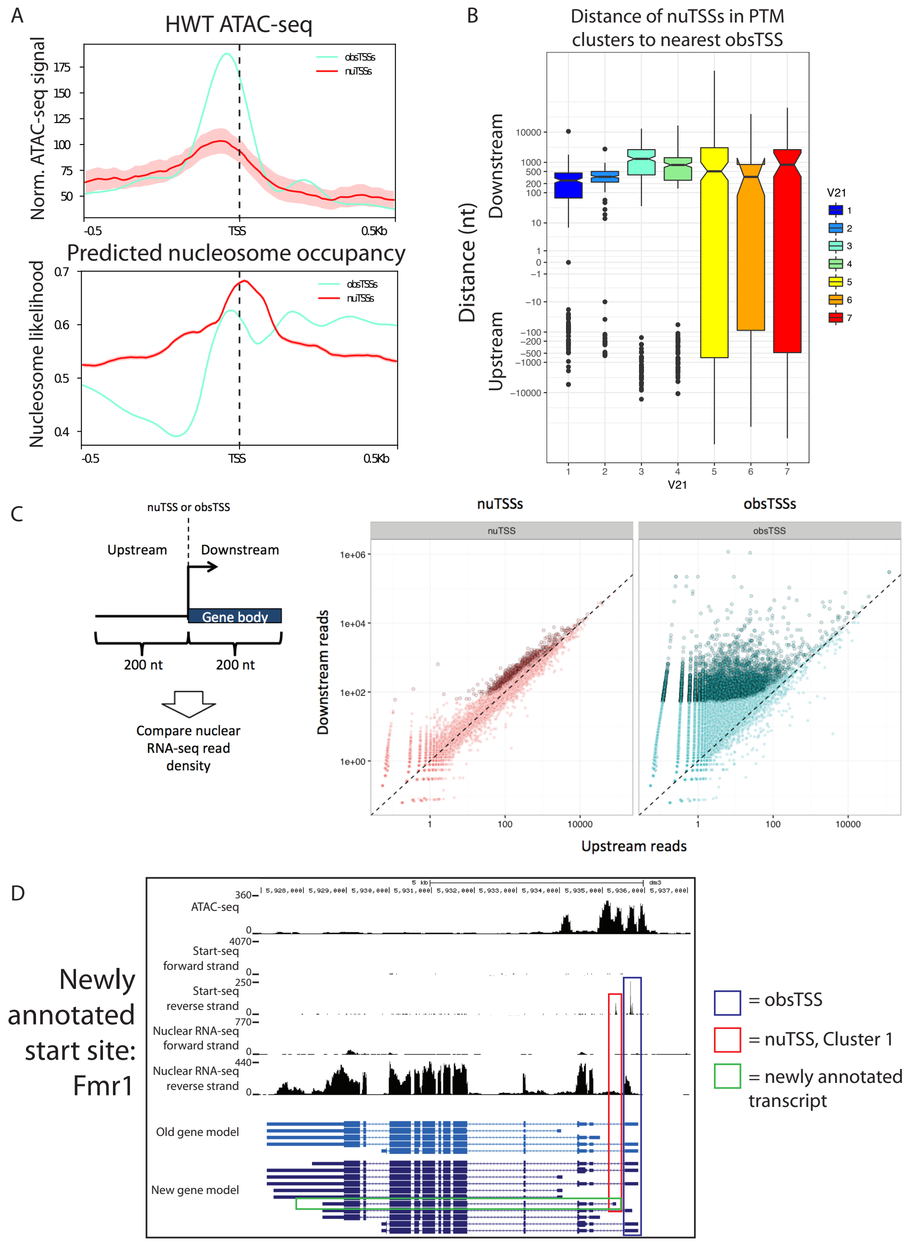

Figure S6: A) Metaplots describing ATAC-seq signal (top) and predicted nucleosome occupancy (bottom) in a 1 kb window surrounding obsTSSs (teal) or nuTSSs (red). B) Boxplot describing distribution of distances from the nearest obsTSS for all nuTSSs in each of 7 histone PTM-defined clusters described in Figure 5A. Negative values indicate nuTSSs that are upstream of the nearest obsTSS, while positive values indicate nuTSSs that are downstream of the nearest obsTSS. C) Nuclear RNA-seq reads were mapped to regions 200nt upstream and downstream of each TSS, and enrichment of downstream vs. upstream reads was analyzed as a proxy for elongation. At right: scatterplots of nuclear RNA-seq upstream reads (X-axis) vs. downstream reads (Y-axis) for nuTSSs (left, salmon) and obsTSSs (right, teal). D) Representative example of a nuTSS belonging to Cluster 1 (red box) that corresponds to a TSS that has been newly annotated in the latest *D. melanogaster* genome build (green box).

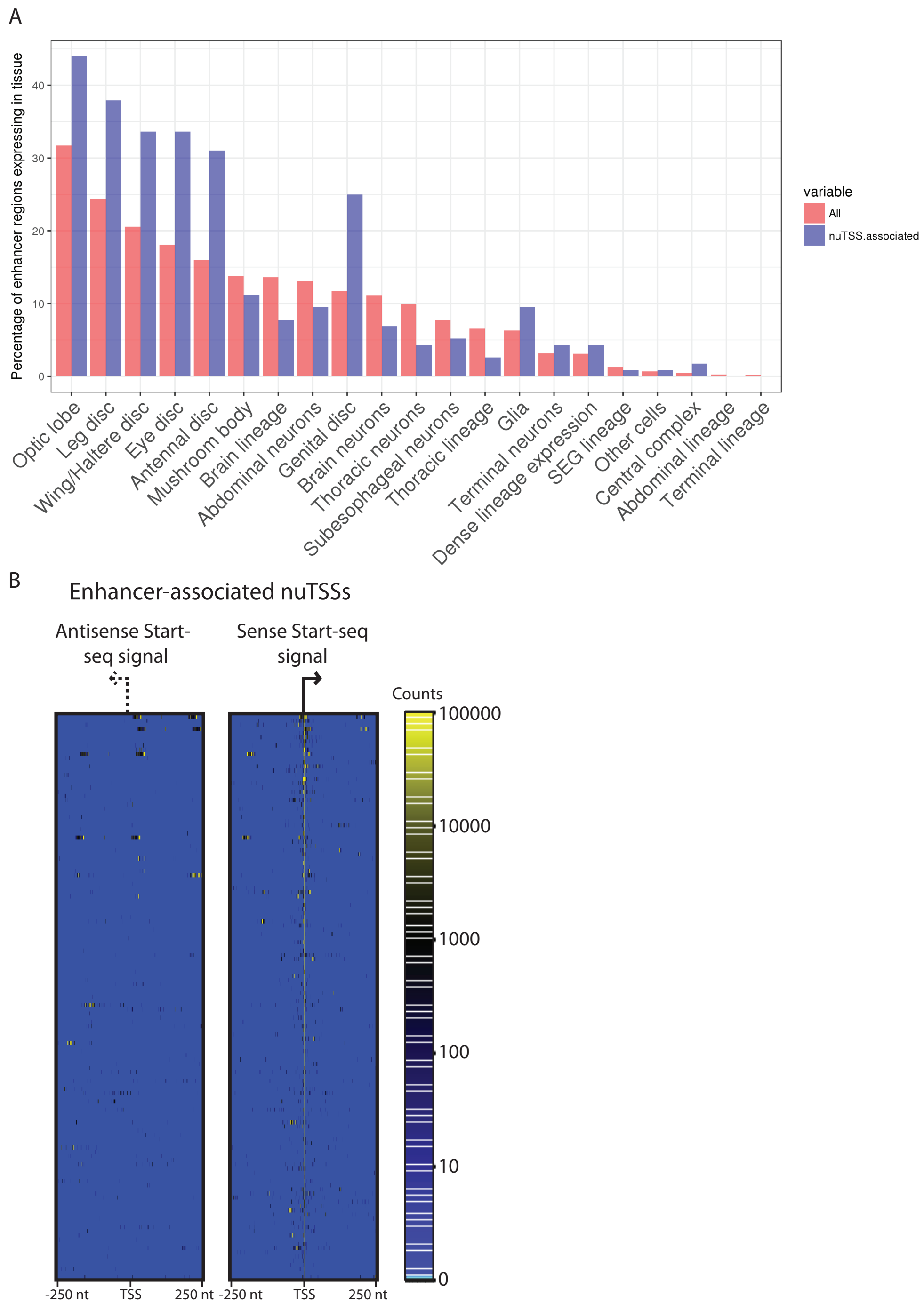

Figure S7: A) Percentage of all FlyLight enhancers (red) or those overlapping with a high-confidence nuTSS (blue) that correspond to each of several larval expression categories. B) Heatmap describing sense (right) or antisense (left) Start-seq signal mapping in a 500nt window around enhancer-associated nuTSSs.
